# Supplementary material for: Influence of Laminin Coating on the Autologous In Vivo Recellularization of Decellularized Vascular Protheses
Source: Materials (Basel). 2019 Oct 15;12(20):3351. doi: 10.3390/ma12203351 (PMC6829566; doi:10.3390/ma12203351)
Supplement: Supplementary file 1 [file materials-12-03351-s001.pdf]

materials

Article

Influence of Laminin Coating on the Autologous In Vivo Recellularization of Decellularized Vascular Protheses

Mahfuza Toshmatova, Sentaro Nakanishi, Yukiharu Sugimura, Vera Schmidt, Artur Lichtenberg, Alexander Assmann *,† and Payam Akhyari

Department of Cardiovascular Surgery and Research Group for Experimental Surgery, Medical Faculty, Heinrich Heine University, Düsseldorf 40225, Germany; Mahfuza.Toshmatova@med.uni-duesseldorf.de (M.T.); snakanishi@asahikawa-med.ac.jp (S.N.); Yukiharu.Sugimura@med.uni-duesseldorf.de (Y.S.); Vera.Schmidt@med.uni-duesseldorf.de (V.S.); Artur.LIchtenberg@med.uni-duesseldorf.de (A.L.); payam.akhyari@med.uni-duesseldorf.de (P.A.)

† Further affiliation beyond this work: Biomaterials Innovation Research center, Brigham and Women´s Hospital, Department of Medicine, Harvard Medical School, Boston, MA 02139, USA

* Correspondence: alexander.assmann@med.uni-duesseldorf.de; Tel.: +49-211-81-18331

Received: 18 August 2019; Accepted: 10 October 2019; Published: date

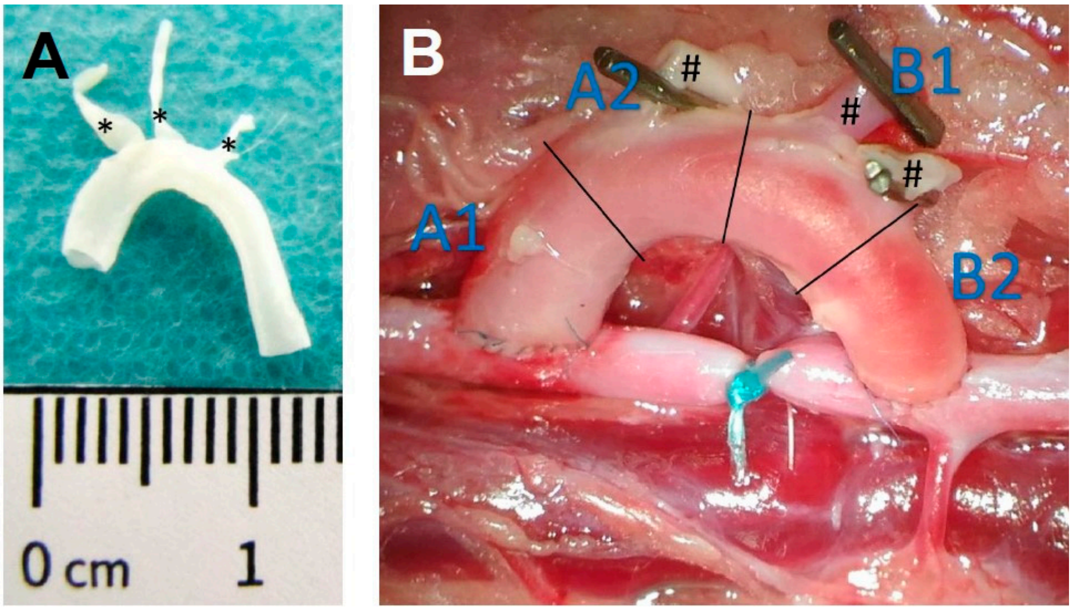

Supplemental Figure S1: Decellularized aortic graft after laminin coating (**A**, asterisks indicate supraaortic branches). Decellularized graft after implantation to the infrarenal aorta (**B**, hashs indicate supraaortic branches, **A1**–**B2** indicate the four graft regions defined for read-out after explantation).

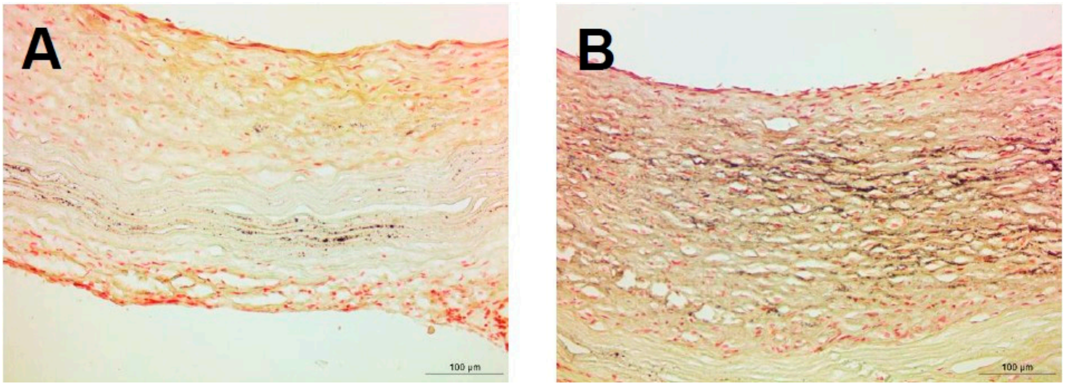

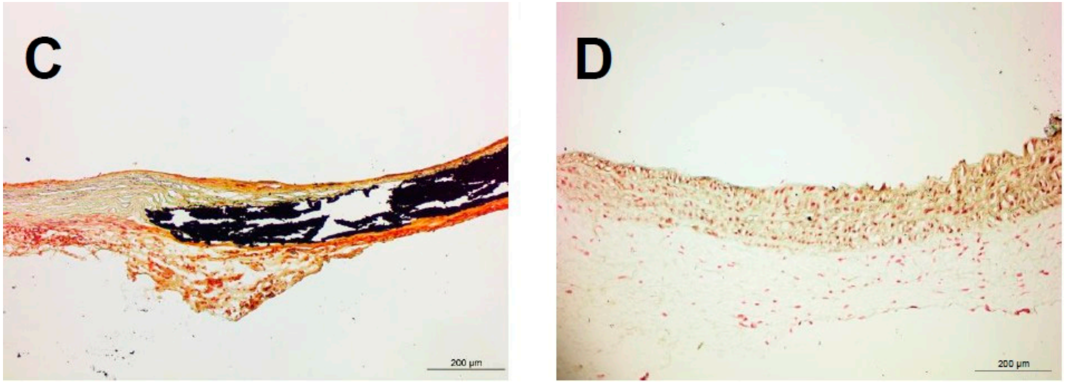

Supplemental Figure S2: Representative cross-sections through the ascending aorta of grafts after 8 weeks *in vivo*. In both groups, minor local (**A**) or extended (**B**) microcalcifications and macrocalcifications (**C**) were observed. Medially repopulated areas did not show any calcifications (**D**). Von Kossa staining. Scale bars = 100 µm in A,B / 200 µm in **C**,**D**.
